# A Prototype Distributed Six-Port Reflectometer-Based Transmission-Only Vector Network Analyzer

**DOI:** 10.3390/s26165218

**Published:** 2026-08-18

**Authors:** Muhammad Hashir, Colin Gilmore, Joe LoVetri

**Affiliations:** Electrical and Computer Engineering Department, University of Manitoba, Winnipeg, MB R3T 2N2, Canada

**Keywords:** six-port reflectometer, calibration, distributed transmission-only vector, network analyzer

## Abstract

A novel distributed Transmission-Only Vector Network Analyzer (TVNA) based on two Six-Port Reflectometers (SPRs) is experimentally verified. The prototype *distributed* SPR-TVNA measures $S_{21}$ and operates with two independent RF sources, allowing the two ports of the Device Under Test (DUT) to be widely separated with no common cables coming to a central location. Using the distributed SPR-TVNA allows for measurements of, e.g., distantly separated antennas, or taking grain bin imaging measurements that would otherwise require long coaxial cables. A key issue of these large DUTs is accurate measurement of the through-measurement, $S_{21}$. The distribution (separation) of the two RF sources in the SPR-VNA requires novel procedures to obtain accurate results. The results are confirmed using simulations and experiments, with the experimental results obtained using custom-built hardware. While, in theory, a full VNA can measure $S_{11}$ and $S_{22}$, which is confirmed through simulation, the experimental results show significant errors in $S_{11}$ and $S_{22}$. We thus call the current device a Transmission-Only VNA. The fundamental math of the SPR-VNA means there is a ±180° phase uncertainty that must be solved, e.g., via a wide-band sweep looking for phase jumps. The experimental $S_{21}$ measurements of the prototype show maximum errors of 1.5 dB in magnitude and 6 degrees in phase over a bandwidth of 0.6–1.2 GHz.

## 1. Introduction

The accurate characterization of radio frequency (RF) and microwave (MW) devices and subsystems is essential for designing and characterizing the performance of complex networks. Vector Network Analyzers (VNAs) are the standard measurement tool. Using the VNA, one typically measures the full *S*-parameter matrix (magnitude and phase) that characterizes the two-port system. For accurate phase measurements, VNAs incorporate a phase reference requiring access to all ports of the Device Under Test (DUT). Of course, calibration techniques are commonly utilized to bring the *S*-parameters’ measurement planes to the ends of the cables connecting the VNA ports to the DUT ports (e.g., [1]).

For certain measurements, the DUT is physically very large, and its ports are distantly separated, making the use of cables from the DUT ports to the VNA not only very cumbersome but also detrimental to achieving accurate measurements. The dynamic range is compromised because of the cable loss, and the phase accuracy deteriorates because of movement in the cables (e.g., due to wind). Examples of these scenarios include grain bin imaging [2,3,4], geophysical applications [5], or measuring 3D MIMO channels [6]. For such large DUTs with distantly separated ports, the need for cables connecting the VNA to the DUT ports can be a serious problem. For example, in the grain bin imaging application, cable lengths of more than 100 m can be required, and cable losses in the two cables can approach more than 60 dB. Moreover, the requirement for a large number of antennas (e.g., 24-port DUTs in the system described in [2,3,7]) means added costs and installation complications for the measurement setup. By far, the most difficult measurement for these large DUTs is the through-measurement $S_{21}$, and in the grain bin application, only $S_{21}$ is used for inversion [2,4]. Thus, accurate measurement of $S_{21}$ is the key parameter of interest.

Beyond the cable problems with physically large DUTs, microwave/electromagnetic imaging has a wide variety of biomedical applications [8,9,10,11,12,13,14,15]. In these applications, VNAs are connected through shorter cables to a series of antennas around the patient. Many coaxial cables, when moved, have small changes in their internal parameters (particularly as regards the phase). The sensitivity required, e.g., to detect the field changes caused by a hemorrhagic, ischemic stroke, or a breast tumor are very small compared to the direct wave between antennas. The changes in S-parameters due to cable movement can easily be on the order of the medically relevant structures. The use of a cable-free measurement system could completely eliminate this source of error.

**Figure 1:**
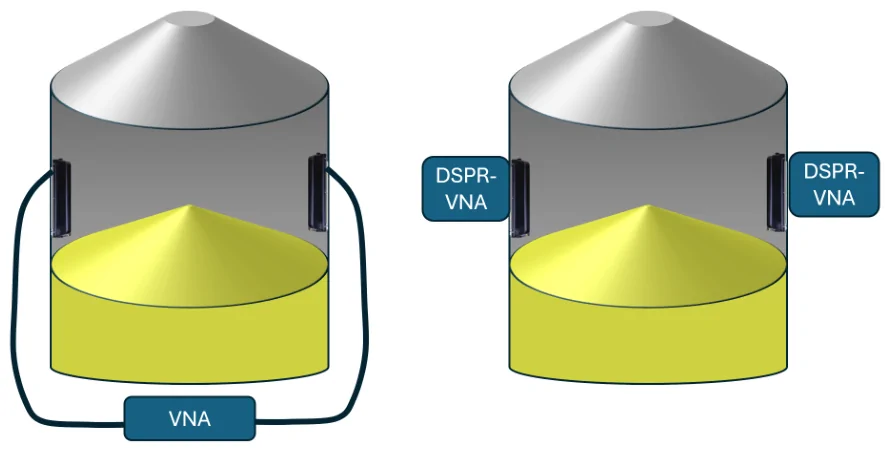
Simplified schematic image motivating a distributed VNA measurement for a grain bin imaging example. The long RF cables required to reach each antenna result in large signal losses and difficulty with maintaining calibration accuracy. By using a distributed SPR-VNA, we can eliminate the RF cables.

Motivated by these applications, we introduce the first reduction to practice of a *distributed* VNA based on two six-port reflectometers (SPRs), which requires no cables connecting the two ports of the DUT. In theory, our device is able to measure both the magnitude and phase of the full *S*-parameter matrix characterizing the propagation path between the two ports. In practice, the current device is inaccurate for reflection measurements $S_{11},S_{22}$. However, reasonable accuracy is achieved for the $S_{21}$ measurement. The current device could thus be called a Transmission-Only VNA (TVNA). An example of the distributed SPR-(T)VNA concept for a grain bin imaging application is shown in Figure 1. Thus, using two disconnected measurement systems, placed at each end of the DUT, the $S_{21}$-parameter of the propagation path can be measured without incurring large cable losses and phase errors. In addition, the proposed design extends to as many ports as required.

Each SPR in the distributed TVNA requires its own RF/MW signal source but these do not need to be synchronized with respect to their phase. In a typical SPR setup [16], the DUT is attached to one of the six ports and an RF source to another port. At each frequency of interest, the *scalar* power transferred to a matched load is measured at the remaining four ports. From these four power measurements, one obtains both the magnitude and phase of the DUTs reflection coefficient, $\Gamma_{DUT}$ (see, e.g., [16,17]). The process of going from four scalar power measurements to the reflection coefficient is non-linear, and many algorithms with associated calibration techniques have been proposed to solve this problem in the past, e.g., [17,18,19]. Six port networks have seen many applications, with a focus on the low-cost of their hardware, e.g., [20,21]. A review on applications of six-port networks can be seen in [22].

In 1977, Hoer [23] proposed the design of a VNA based on the use of two SPRs. By using two SPRs and connecting them with a single RF source, Hoer showed that by modifying the phase of the RF source going to one of the ports, and taking a series of SPR measurements of just the reflection parameters at each port, ($\Gamma_{DUT,1}$ and $\Gamma_{DUT,2}$), one can obtain the full two-port *S*-matrix of the DUT. This technique [23] can be called an SPR-based VNA (or SPR-VNA). This SPR-VNA technique requires phase and/or amplitude shifters to modify the RF source to one of the ports. While not in widespread use, work on SPR-VNAs continues for some applications, e.g., [24], and similar ‘measure only power’ concepts for one-port VNAs have been built [25,26].

In this work, we implement a novel extension of the SPR-VNA, with the key contribution that two separated, non-phase-locked, RF sources are used for each SPR (located right at the port of the DUT). This was first conceptualized in [27]. This paper represents the first experimental prototype of such a device and is an extension of the thesis work in [28]. While the synthetic results here show the capability of a full two-port VNA, the experimental results are accurate only for Transmission-Only. Henceforth, we use the term SPR-TVNA for the experimental device, and SPR-VNA for the theoretical full two-port device.

This paper is organized as follows: In Section 2 we provide the relevant background on the SPR that will be used in describing the SPR-VNA, including the non-linear optimization algorithm we use to solve for the reflection coefficient at the DUT port of the SPR. In Section 3, we review the standard SPR-VNA theory. Section 4 introduces the distributed SPR-VNA theory, followed by our particular implementation of the DSPR-TVNA in Section 5. The unique experimental measurement technique is covered in Section 6, with simulated and experimental verification reported in the following two sections. A discussion of the results and the conclusions follow.

## 2. Background on Six-Port Reflectometers

Before we can describe a distributed SPR-VNA (used to obtain $S_{21}$ of the DUT), it is important to explore the basics of six-port reflectometers (used to obtain reflection coefficients only) and highlight the methods used to perform the SPR calculations. The accuracy of the SPR is critical to the extension to an SPR-VNA.

**Figure 2:**
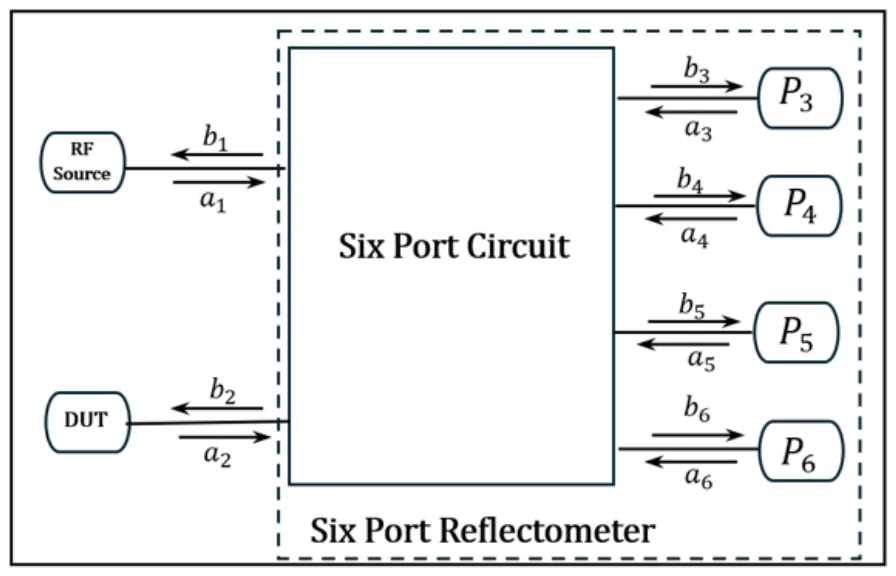
Block diagram of a six-port reflectometer.

The SPR, which evolved from the ‘Six-Port Correlator’ [17], is a circuit comprising six ports, with four of these ports equipped with external power detectors. A block diagram of an SPR is shown in Figure 2. One port is connected to a primary RF source (a device outputting a sinusoidal wave voltage at a particular frequency), while the second port interfaces with the DUT. Theoretically, a six-port circuit (sometimes referred to as a six-port junction) that is used to create a SPR can have virtually any internal topology, but certain topologies have better properties for stable measurements [17].

The six-port circuit can be characterized using a scattering matrix, $S^{6}$, which relates the incident voltage waves (referred to as *a*), and the reflected voltage waves (*b*) at a given frequency and each port [1] (We use the ‘6’ in $S^{6}$ to distinguish the six-port circuit *S* matrix from other *S*-matrices used throughout this work). In matrix form:

(1) $$\underline{b}=S^{6}\underline{a}$$

where $\underline{a}=\left[a_{1};a_{2};\ldots a_{6}\right]$ and $\underline{b}=\left[b_{1};b_{2};\ldots b_{6}\right]$ are vectors of the incident and reflected waves at each port. The ratio of incident to the reflected voltage waves at each port is the reflection coefficient $\left(\Gamma_{i}\right)$:

(2) $$\Gamma_{i}=\frac{a_{i}}{b_{i}}\;\;\mathrm{where}\;i=1\ldots 6$$

To use this six-port circuit as an SPR, we attach an RF source to port 1, and the DUT to port 2, and then attach four power meters at ports 3–6, as in Figure 2. Using this setup, the goal of the remaining SPR analysis is to use the power measurements ($|b_{i}{|}^{2}$) at ports 3 to 6 to obtain the reflection coefficient of the DUT at port 2. That is, our unknown is $\Gamma_{2}=\Gamma_{DUT}$, and our knowns are $P_{i}={\left|b_{i}\right|}^{2}$ at the measurement ports.

### 2.1. SPR Theory

The process of using the power measurements from the SPR to determine $\Gamma_{DUT}$ is well documented in the literature, e.g., [16,17,23,29,30,31]. Here, we give an overview of the most common methods of SPR analysis. Performing a review of SPR analysis is beyond the scope of this paper, and interested readers can see the references.

The process of turning four power measurements into $\Gamma_{DUT}$ begins by substituting $a_{i}=\Gamma_{i}b_{i}$ at ports i=3…6 inside of Equation (1). After performing some simple manipulations, and assuming non-ideal power-detectors, we end up with the matrix shown in Equation (3):

(3) $$\left[\begin{array}{c} b_{1}\\ b_{2}\\ 0\\ 0\\ 0\\ 0 \end{array}\right]=\left[\begin{array}{cccccc} S_{11} & S_{12} & S_{13}\Gamma_{3} & S_{14}\Gamma_{4} & S_{15}\Gamma_{5} & S_{16}\Gamma_{6}\\ S_{21} & S_{22} & S_{23}\Gamma_{3} & S_{24}\Gamma_{4} & S_{25}\Gamma_{5} & S_{26}\Gamma_{6}\\ S_{31} & S_{32} & S_{33}\Gamma_{3}-1 & S_{34}\Gamma_{4} & S_{35}\Gamma_{5} & S_{36}\Gamma_{6}\\ S_{41} & S_{42} & S_{43}\Gamma_{3} & S_{44}\Gamma_{4}-1 & S_{45}\Gamma_{5} & S_{46}\Gamma_{6}\\ S_{51} & S_{52} & S_{53}\Gamma_{3} & S_{54}\Gamma_{4} & S_{55}\Gamma_{5}-1 & S_{56}\Gamma_{6}\\ S_{61} & S_{62} & S_{63}\Gamma_{3} & S_{64}\Gamma_{4} & S_{65}\Gamma_{5} & S_{66}\Gamma_{6}-1 \end{array}\right]\left[\begin{array}{c} a_{1}\\ a_{2}\\ b_{3}\\ b_{4}\\ b_{5}\\ b_{6} \end{array}\right]=\bar{S^{6}}\left[\begin{array}{c} a_{1}\\ a_{2}\\ b_{3}\\ b_{4}\\ b_{5}\\ b_{6} \end{array}\right]$$

where $S_{xy}$ denotes the matrix elements of $S^{6}$. By noting in Equation (3) that the modified $S^{6}$ matrix is denoted $\bar{S^{6}}$, we can define its inverse as follows:

(4) $$M={\left(\bar{S^{6}}\right)}^{-1}.$$

By letting each element of *M* be denoted by $m_{xy}$, we can then use Equation (3) to write:

(5) $$\begin{array}{ccc} a_{1} & = & m_{11}b_{1}+m_{12}b_{2} \end{array}$$

(6) $$\begin{array}{ccc} a_{2} & = & m_{21}b_{1}+m_{22}b_{2} \end{array}$$

(7) $$\begin{array}{ccc} b_{i} & = & m_{i1}b_{1}+m_{i2}b_{2} \end{array}$$

for i=3…6. Through a series of lengthy but straightforward algebraic manipulations [28], we end up with the set of equations:

(8) $$\frac{P_{i}}{P_{3}}=\frac{|b_{i}{|}^{2}}{|b_{3}{|}^{2}}=\frac{|m_{i1}{|}^{2}{\left(\Gamma_{2}-q_{i}\right)}^{2}}{|m_{31}{|}^{2}{\left(\Gamma_{2}-q_{3}\right)}^{2}}\;i=4\ldots 6,$$

where $P_{i}={\left|b_{i}\right|}^{2}$ is the power measurement, and $q_{i}=\frac{m_{i2}m_{21}}{m_{i1}}$. We can re-write Equation (8) as follows:

(9) $${\left|\Gamma_{DUT}-q_{3}\right|}^{2}k_{i}={\left|\Gamma_{DUT}-q_{i}\right|}^{2}\;i=4\ldots 6,$$

where $k_{i}=\frac{P_{i}m_{31}}{P_{3}m_{i1}}$.

### 2.2. Solving the Non-Linear SPR Problem

The three equations represented in Equation (9) (or (8)) are the key to going from power measurements to the unknown reflection coefficient, and there are many ways to frame this set of equations. Equation (9) can be considered as a non-linear problem with three equations and two unknowns (real and imaginary part of $\Gamma_{DUT}$). One can then solve these equations for $\Gamma_{DUT}$ with many methods.

However, in SPR literature, it is common [17] to frame (9) as a series of three circles on the real/imaginary plane, with centers $q_{i}$, and radii of $R_{i}^{2}={\left|\Gamma_{DUT}-q_{3}\right|}^{2}k_{i}$. The centers of the circles, $q_{i}$ are functions of the six-port-circuit ($S^{6}$) and power meter reflection coefficients, while the radii are functions of $\Gamma_{DUT}$, the power measurements, $S^{6}$ and the power meter reflection coefficients. The true $\Gamma_{DUT}$ lies at the intersection of the three circles. The design of a successful SPR also depends on appropriately distributing the $q_{i}$ points evenly in the real/imaginary plane [17]. In practice, measurement noise and other errors means that there is no $\Gamma_{DUT}$ that perfectly satisfies all three equations, and optimal solutions must be sought.

By expanding the squared quantities in (9), and then performing more manipulations, one can arrive at ([28], Section 2.4):

(10) $$|\Gamma_{DUT}-C_{i}{|}^{2}=R_{i}^{2}\;i=4\ldots 6$$

where:

(11) $$\begin{array}{c} C_{i}=\frac{q_{3}k_{i}-q_{i}}{k_{i}-1} \end{array}$$

(12) $$\begin{array}{c} R_{i}=\frac{\sqrt{k_{i}}}{|k_{i}{-1|}^{2}}\left|q_{i}-q_{3}\right|. \end{array}$$

Equation (10) still has the geometric interpretation of three circles in the real/imaginary plane of $\Gamma_{DUT}$ but removes the direct dependence of the radii on $\Gamma_{DUT}$. Following [17,31], we use Equation (10) throughout this work.

While there are many possibilities for solving (10), here, we use the Gauss–Newton method [32]. We treat the real and imaginary parts of $\Gamma_{DUT}$ as two real-valued unknowns and minimize the objective function:

(13) $$\min_{\Gamma_{DUT}}F\left(\Gamma_{DUT}\right)=\sum_{i=1}^{3}{\left(R_{i}^{2}-{\left(\Gamma_{DUT}-C_{i}\right)}^{2}\right)}^{2}$$

The Jacobian matrix *J* is calculated as J1=∂F∂Re(ΓDUT) and J2=∂F∂Im(ΓDUT), which is the first-order partial derivatives of the residual functions with respect to the real and imaginary parts of $\Gamma_{DUT}$. We check for convergence by evaluating the updated $\Gamma_{DUT}$.The convergence level we selected was $\left|\Gamma_{DUT}^{\left(i+1\right)}-\Gamma_{DUT}^{\left(i\right)}\right|<10^{-10}$. If this condition is met, the optimization process stops.

## 3. The Six-Port Reflectometer-Based Vector Network Analyzer

**Figure 3:**
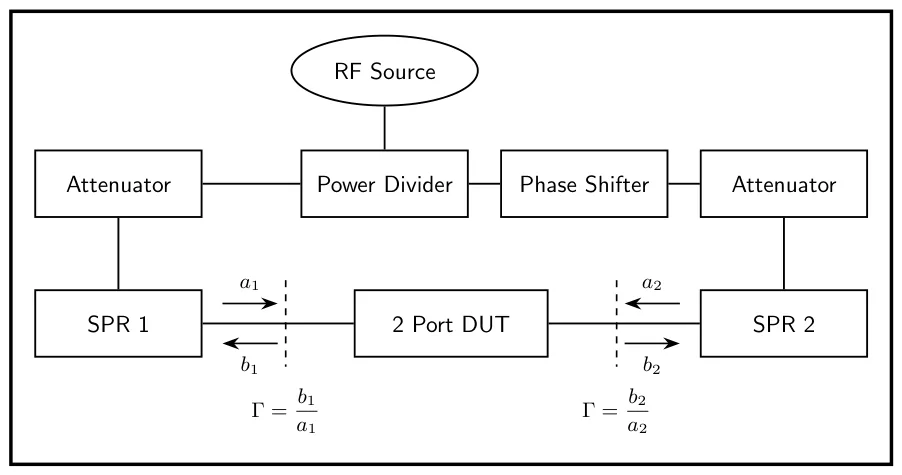
Topology of a six-port network analyzer (SPR-VNA) [23].

We next consider the extension of the SPR into an SPR-VNA. The theory behind the SPR-VNA was first introduced by Hoer [23]. His proposed design is shown in Figure 3. An SPR-VNA employs two SPRs with a single RF source to measure the complex ratios $\Gamma_{1}=b_{1}/a_{1}$ and $\Gamma_{2}=b_{2}/a_{2}$, where $b_{1}$ and $b_{2}$ are the waves emanating from the DUT, while $a_{1}$ and $a_{2}$ are the waves impinging on the DUT. These ratios are related to the (two-port) S-parameters of the DUT via the shematic shown below.

(14) $$\begin{array}{cc} & b_{1}=S_{11}a_{1}+S_{12}a_{2}, \end{array}$$

(15) $$\begin{array}{cc} & b_{2}=S_{21}a_{1}+S_{22}a_{2}. \end{array}$$

Dividing (14) by $a_{1}$ and (15) by $a_{2}$ and using $\Gamma_{1}=\frac{b_{1}}{a_{1}}$ and $\Gamma_{2}=\frac{b_{2}}{a_{2}}$ gives:

(16) $$\begin{array}{cc} & \Gamma_{1}=S_{11}+S_{12}\frac{a_{2}}{a_{1}}, \end{array}$$

(17) $$\begin{array}{cc} & \Gamma_{2}=S_{22}+S_{21}\frac{a_{1}}{a_{2}}. \end{array}$$

Adding (16) and (17), we get:

(18) $$\begin{array}{c} \Gamma_{1}+\Gamma_{2}=S_{11}+S_{22}+S_{12}\frac{a_{2}}{a_{1}}+S_{21}\frac{a_{1}}{a_{2}}. \end{array}$$

If we multiply (16) with $\Gamma_{2}$ on both sides:

(19) $$\Gamma_{1}\Gamma_{2}=\left(S_{11}+S_{12}\frac{a_{2}}{a_{1}}\right)\Gamma_{2}.$$

Using (17), we can replace $\Gamma_{2}$ in (19), and performing some algebra, we can arrive at:

(20) $$\Gamma_{1}\Gamma_{2}=S_{11}\Gamma_{2}+S_{22}S_{12}\frac{a_{2}}{a_{1}}+S_{12}S_{21}$$

If we multiply (16) by $S_{22}$ on both sides, we get:

(21) $$\Gamma_{1}S_{22}=S_{11}S_{22}+S_{22}S_{12}\frac{a_{2}}{a_{1}}$$

Subtracting (21) from (20), and simplifying, we can arrive at:

(22) $$\Gamma_{1}\Gamma_{2}=\Gamma_{1}S_{22}+\Gamma_{2}S_{11}-\Delta ,$$

where:

(23) $$\Delta =\left|\begin{array}{cc} S_{11} & S_{12}\\ S_{21} & S_{22} \end{array}\right|=S_{11}S_{22}-S_{21}S_{12}.$$

Equation (22) is the key equation of SPR-VNAs. It is an equation in three complex-valued unknowns: $S_{11},S_{22}$, and Δ, where $\Gamma_{1}$ and $\Gamma_{2}$ depend on both the DUT and (critically) $a_{1}$ and $a_{2}$ waves (In microwave engineering, it is common to equate $\Gamma_{1}$ with $S_{11}$, but they are only equal when there is a single source at port 1, and all other sources are zero. With two sources, $\Gamma_{1}\neq S_{11}$). Using the SPRs and theory outlined in Section 2, we can obtain $\Gamma_{1}$ and $\Gamma_{2}$ for any DUT and any set incoming waves. We can next turn (22) into a *system* of equations by changing the magnitude and/or phase of $a_{1}$ or $a_{2}$ (as shown in Figure 3). By changing the incoming waves, we change $\Gamma_{1}$ and $\Gamma_{2}$, while the DUT *S*-parameters remain constant. Thus, Equation (22) can represent a system of equations in the three (complex) unknowns: $S_{11},S_{22}$ and Δ. Assuming that we use the phase shifter (and possibly an attenuator) in Figure 3, to change $a_{1}$, and denoting the different reflection coefficients with respect to this RF source change as $\Gamma^{\theta_{i}}$, we can write (22) as a matrix equation for our three unknowns:

(24) $$\left[\begin{array}{ccc} \Gamma_{2}^{\theta_{1}} & \Gamma_{1}^{\theta_{1}} & -1\\ \Gamma_{2}^{\theta_{2}} & \Gamma_{1}^{\theta_{2}} & -1\\ \Gamma_{2}^{\theta_{3}} & \Gamma_{1}^{\theta_{3}} & -1 \end{array}\right]\left[\begin{array}{c} S_{11}\\ S_{22}\\ \Delta \end{array}\right]=\left[\begin{array}{c} \Gamma_{1}^{\theta_{1}}\Gamma_{2}^{\theta_{1}}\\ \Gamma_{1}^{\theta_{2}}\Gamma_{2}^{\theta_{2}}\\ \Gamma_{1}^{\theta_{3}}\Gamma_{2}^{\theta_{3}} \end{array}\right]$$

To guarantee this matrix has a reasonable condition number, the changes in $a_{1}$ (and/or $a_{2}$) must change the Γs sufficiently so the matrix has linearly independent rows. It is further possible to use even more shifts in the *a*s to create a larger matrix system, and solve (24) using least squares.

Once $S_{11},S_{22}$, and Δ are obtained via (24), we still need to determine $S_{12},S_{21}$. Here, we assume a reciprocal network ($S_{12}=S_{21}$) (It should be noted that Hoer provides methods of using SPR-VNAs for non-reciprocal networks [23] as well, but we only consider reciprocal networks). Then, by using (23), we can write:

(25) $$\begin{array}{c} {\left|S_{12}\right|}^{2}={\left|S_{21}\right|}^{2}={\left|S_{11}S_{22}-\Delta \right|}^{2} \end{array}$$

(26) $$\begin{array}{c} S_{12}=S_{21}=\pm \sqrt{S_{11}S_{22}-\Delta } \end{array}$$

From (26), we are thus able to use our knowledge of $S_{11}$, $S_{22}$ and Δ to determine $S_{21}$.

### Phase Uncertainty

Equation (26) also places fundamental limits on the SPR-VNA. The ± in front of the calculation does not affect the magnitude of our $S_{21}$ calculation, but means that we can only ever know the phase within a ± π (rad) (or ±180 deg) accuracy. In practice for wide-band systems, this means that the phase can wrap with jumps of π. Hoer suggests [23] that the choice of the plus or minus must be made with prior knowledge of the expected value (as shown here, for multi-frequency systems, one can unwrap phase jumps on the order of π rad). Here, we select the initial (first frequency) phase via knowledge from a direct measurement with a regular VNA. Further, (26) shows that errors in $S_{11}$ and $S_{22}$ can cancel out, and result in accurate $S_{21}$ calculations even through $S_{11}$ and $S_{22}$ are incorrect. This will again be seen in our results (Section 8.3).

## 4. The Distributed SPR-VNA

**Figure 4:**
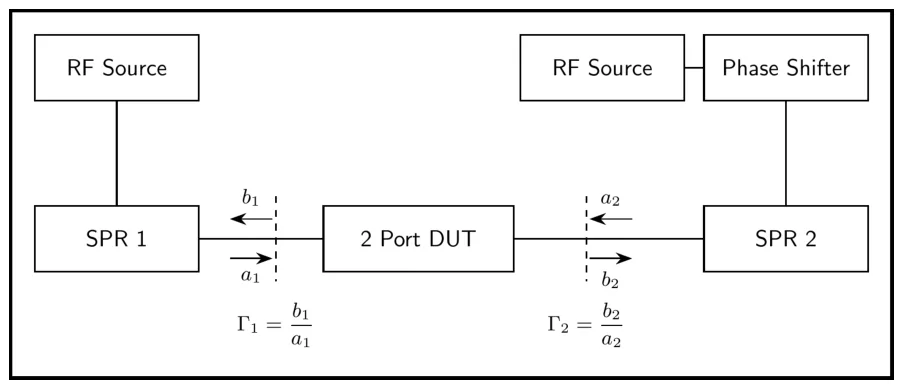
Proposed design of a distributed SPR-VNA, with two separated RF Sources.

The key realization to create a distributed SPR-VNA (or DSPR-VNA)—and eliminate the cables needed for a typical VNA measurement—is that the mathematical foundations of the SPR-VNA discussed above do not require a single RF source. The only requirement is that we need to vary (at least one of) the incoming waves to one of the two DUT ports, $a_{1}$ and $a_{2}$, and take a measurement each time with both SPRs. Changing the incoming RF source changes the Γs and thus allows us to solve Equation (24). There is no need to have two phase-linked sources; the only requirement is that several different *a*s be used on one of the ports. Thus, one can completely separate the two RF sources, as per the distributed SPR-VNA design shown in Figure 4. By separating the RF sources into two independent units, with one side of the design containing a phase shifter, we can, in theory, create a full two-port VNA that measures without any direct connection between each SPR (as seen in our results, in practice, this system is only accurate for $S_{21}$); so, it is called a TVNA.

The concept of such a DSPR-(T)VNA was first proposed and simulated within our research group by Aboud [27]. However, ref. [27] presented only simulated results and did not show experimental verification of the DSPR-TVNA. Below, we explore the experimental verification of a DSPR-TVNA.

## 5. Design of the Distributed SPR-VNA

A distributed SPR-VNA (DSPR-VNA) is built around two individual SPRs. Thus, the design of the DSPR-VNA relies on the design of the SPRs used. For this initial prototype DSPR-TVNA, we have selected a frequency range of 0.6 to 1.2 GHz, based primarily on the availability of components and the time required to obtain measurements. An SPR requires the design/selection of a six-port circuit, power detectors, analog-to-digital conversion, and RF sources. Each of our design choices is outlined below.

### 5.1. Six-Port-Circuit Design

While the theory outlined in Section 2 is agnostic to the particulars of the $S^{6}$ matrix, in practice, there are clearly better and worse six-port circuits. Some important design requirements are to ‘spread out’ the centers of each circle center (the $q_{i}$ points in Equation (9), which depend only on the design of $S^{6}$), and to have a direct coupling between the RF source to the ‘reference’ port #3 (since, as per Equation (8), all the other power measurements are normalized by $P_{3}$). While a detailed analysis of ideal SPR design is out of the scope of this work, we review a few key points of our chosen design below.

**Figure 5:**
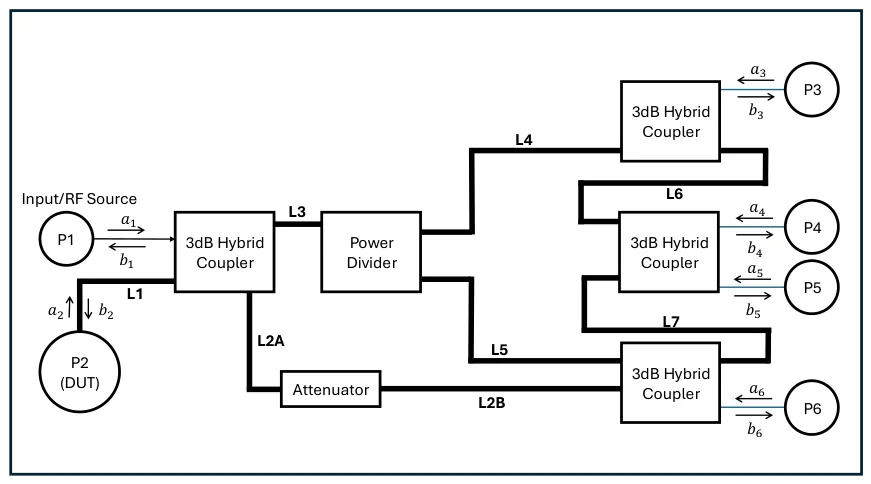
Chosen six-port reflectometer design [27].

**Figure 6:**
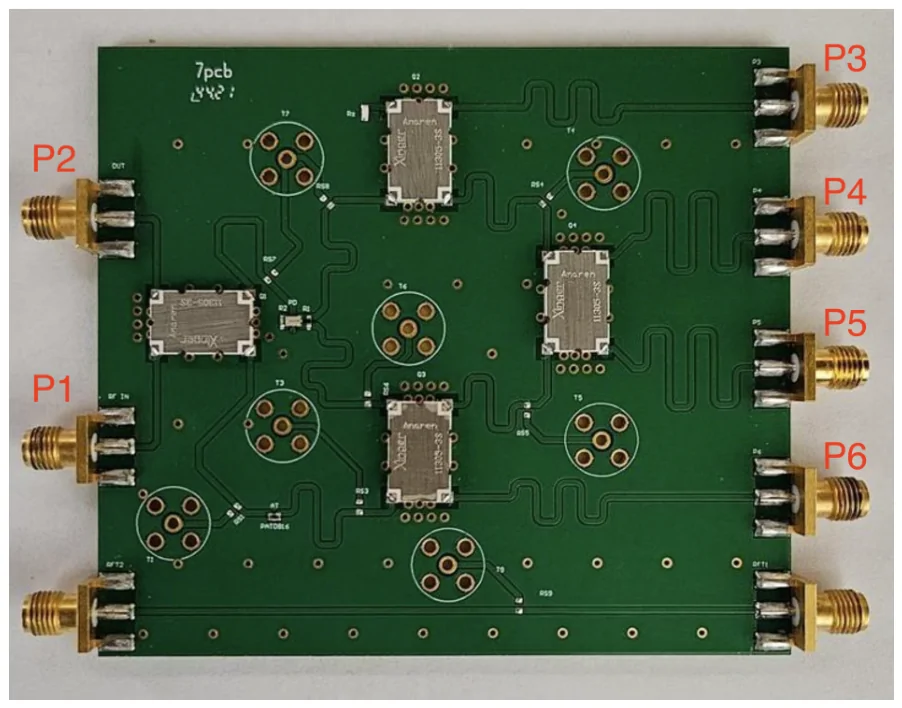
Photograph of our six-port circuit implementation. The 6 ports P1–P6 are labelled in red. Two extra ports (bottom, unlabelled) were added for testing the impedance of the transmission lines to ensure they were 50 Ω, but were not used in this work.

Our study uses a modified version of the frequency compensated quasi-optimal design proposed by Ghannouchi [33]. The schematic of this six-port circuit is shown in Figure 5. Our design was first presented in [27]. The modification from [33] lies in the selection of components and coplanar waveguides used for fabrication of the SPR in our desired frequency range (0.6–1.2 GHz). A photograph of one of the constructed six-port circuits is shown in Figure 6.

For our design, we used 3 dB quadrature hybrid couplers [Anaren model 11035-3S] operating from 1 to 2 GHz, with 0.45 dB insertion loss and 20 dB isolation. The power divider is Anaren model PD0922J5050S2HF. The attenuator is Analog Devices model HMC653LP2E. The connection lines were made using coplanar waveguide transmission lines of 50 ohm impedance and electrical lengths adjusted according to the requirements in [33]. FR-4 substrate with relative permittivity of 4.3 was used.

### 5.2. Power Detectors and ADCs

**Figure 7:**
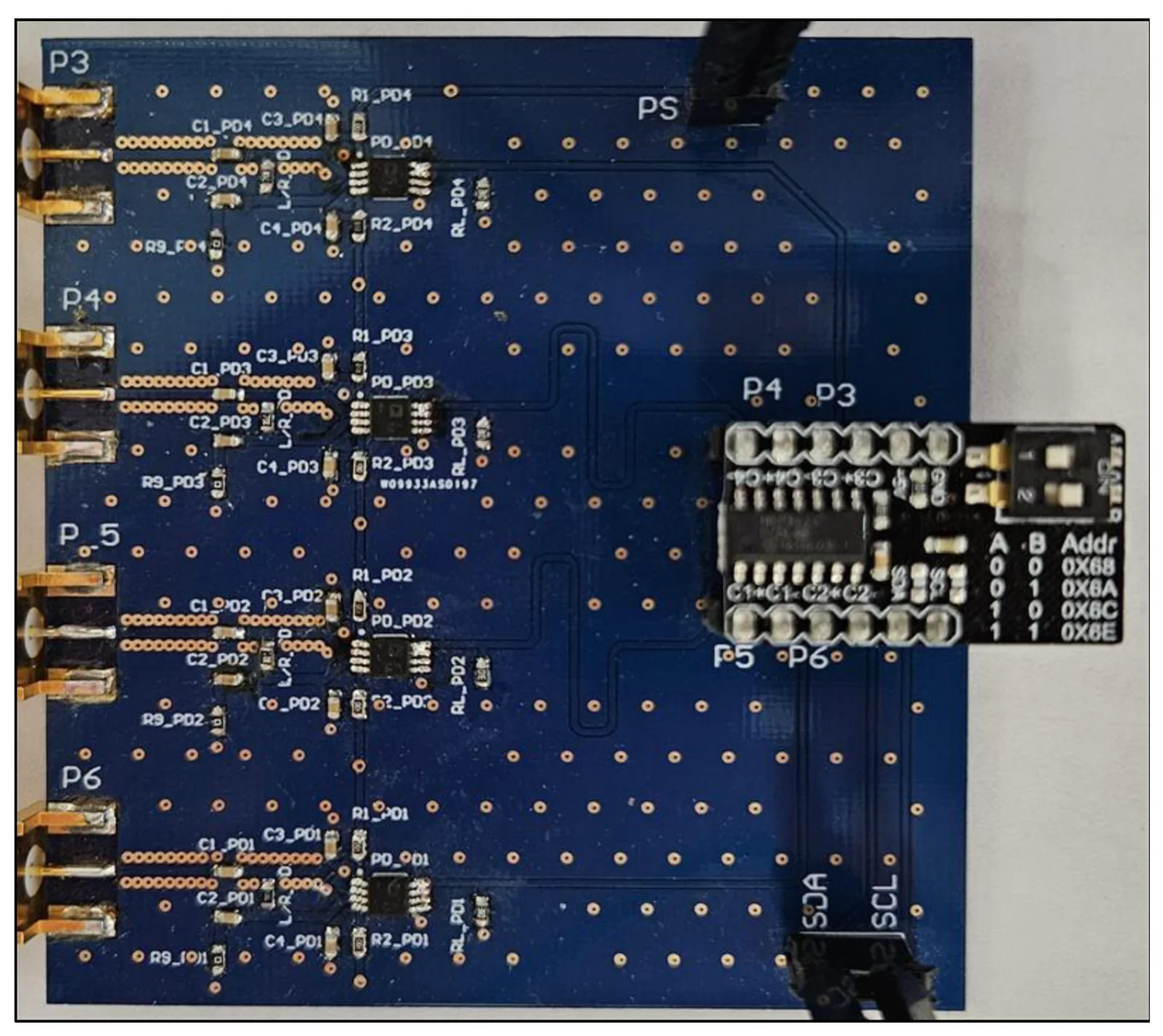
Photograph of the power detector (4 x AD8313) and ADC (MCP3424) board.

For the power detector analog-to-digital circuit (ADC), we developed the board shown in Figure 7. Each of the four required channels uses an Analog Devices AD8313 power detector chip in its standard Log (RSSI) configuration. The output of the power detector is routed to the Microchip Technology MCP3424, a 4-channel, 18-bit ADC converter. One limitation of our ADC is that at 18 bits of resolution, the maximum sample rate is 3.75 samples per second (note that we required the 18 bits of ADC resolution to obtain accurate SPR results). Each ADC was controlled via an Arduino-Uno micro-controller.

### 5.3. RF Sources

For our initial prototype, we selected two lab-bench RF sources: Agilent/Keysight E8267D PSG Vector Signal Generator and Agilent/Keysight N9310 Signal Generator. These are reliable high-frequency sources with a bandwidth from near DC to 20 GHz. The PSG signal generator is capable of adding a phase shift to its output, and this capability was used for the phase shifter in our DSPR-VNA implementation (see Figure 4). Importantly for our results, these two RF sources can link local oscillators if required.

## 6. Our SPR Measurement Technique

The process of using an SPR to measure $\Gamma_{DUT}$ of unknown loads requires characterizing the parameters of the SPR. That is, solving the equations given in (9) or (10) requires not only the power measurements, but also requires knowing, e.g., the $q_{i}$s and $m_{ij}$ values for a given SPR. The process of learning these parameters (or other sets of parameters, depending on the specific SPR mathematical formulation used) is known in the literature as *characterization and calibration* of the SPR, and this process is subject to an extensive literature [16,17,18,19,23,29,30,34,35,36,37,38,39] (to name only a few). This long history and the non-linear nature of the SPR problem (where different formulations can provide different final results) mean that there are many different ways to go from power measurements to the unknown reflection coefficient, $\Gamma_{DUT}$. In this section, we outline the procedure we chose for our SPRs and SPR-VNA procedures but do not provide an extensive review of all possible options.

The most common process of characterization and calibration is to manipulate the core SPR equations (e.g., (9)) to a desired form, and then measure a set of loads with the SPR to learn the underlying parameters of any given six-port-circuit. This process often combines both characterizing the unknown SPR parameters with an error-correction calibration procedure, and the most common characterization/calibration method is a nine-load measurement combined with a ‘6-to-4 port’ reduction, followed by a standard one-port calibration procedure [17,30].

However, with the availability of multi-port VNAs, it is possible to measure the SPR parameters directly. By using a six (or higher)-port VNA, one can simply measure $S^{6}$ of a given SPR, and then measure the reflection coefficients of the power detectors, $\Gamma_{i}$ (i=3…6). This means that all elements of $\bar{S^{6}}$ (Equation (3)) are known, and thus, $M={\left(\bar{S^{6}}\right)}^{-1}$ can readily be computed (assuming a non-singular matrix). Once these values are known, all other parameters, e.g., the $q_{i}$s, can be computed directly.

Given the availability of a multi-Port VNA, we chose the direct measurement method of characterizing the SPR, and use a Keysight (United States) M9802A in a M9019A chassis. The direct measurement method avoids the use of a large number of calibration loads (e.g., nine), and simplifies the data collection procedure. Our experience within lab-based conditions is that the $S^{6}$ and power-detector Γs can be measured once, and then used successfully for more than a year (i.e., the DUT results presented here used measurements of the $S^{6}$ of our boards that were a year old). We do note that this direct measurement may not be possible with some SPR applications (e.g., high power, or high frequency), and alternative methods for characterization/calibration may need to be used.

In summary, by directly measuring $S^{6}$ with a full six-port VNA, we do not need to perform a traditional SPR characterization and calibration. Once we know $S^{6}$, we can directly compute all the needed parameters in Equations (3)–(9).

### 6.1. SOL Calibration and Error Correction

While we were able to obtain reasonable SPR results with the direct measurement of $S^{6}$ and the power detectors, we also found that results could be improved through the use of a standard one-port short-open-load (SOL) calibration [40]. The details of this SOL calibration are given in Appendix A. All experimental measurements shown here used SOL calibration.

### 6.2. Power Detector Calibration

The AD8313 power detector provides an approximately linear output to the input power from −60 dBm to −5 dBm input levels [41]. However, variations in each detector, along with variations in the external matching circuitry, mean that each detector provides slightly different outputs for a given input power. These changes also apply for each frequency. Thus, in addition to measuring the $\Gamma_{i}$s for each port on the device shown in Figure 7, we performed an additional power-detector calibration step.

Each detector in our DSPR-VNA (8 total detectors) was connected directly to our lab-bench RF source. The true output power of our RF source was confirmed with a lab-bench power detector (Agilent/Keysight N1912A (USA)). Then, for frequencies of 0.6 to 1.2 GHz in 1 MHz steps, the output voltage of each power detector was measured with our ADC for input powers ranging from −70 dBm to −5 dBm in 5 dBm steps, providing a voltage-to-dBm conversion by frequency. For each detector, these values were stored, and all subsequent input voltages were converted to input power through linear interpretation of these tables.

### 6.3. SPR Measurement Process Summary

In summary, our SPR characterization, calibration, and measurement process is as follows:

- Characterization measurements performed once (saved and used for many months; these measurements are not required on a daily basis):
  – With multi-port VNA, measure $S^{6}$.
  – With external VNA, measure reflection coefficients of the power detectors $\Gamma_{i}$, i=3…6.
  – Compute $\bar{S^{6}}$ and $M={\left(\bar{S^{6}}\right)}^{-1}$.
  – With a lab-bench RF source, create voltage-to-dBm measurement tables for each power detector.
  – For each SPR, measure known short-open-load one-port loads to compute correction factors.
- For each new DUT:
  – Turn on the RF source and measure the ADC voltages for the DUT.
  – Use the conversion tables from Section 6.2 to convert voltages to power, obtaining $P_{i}$s.
  – Using powers $P_{i}$ and *M*, compute $q_{i}$s and $k_{i}$s from Equations (10)–(12).
  – Use Gauss–Newton optimization to solve for $\Gamma_{DUT}$ in Equation (10).
  – Perform the SOL error correction to correct the measurement.

This process was completed for each desired frequency, and completes a single SPR measurement. For performing an SPR-VNA measurement, the procedure was:

- The two-port DUT was placed between the two SPRs.
- Two different RF sources were turned on (one for each SPR). Both were set to the same output frequency.
- The SPR procedure above was repeated for both SPRs, obtaining $\Gamma_{DUT,1,2}^{\theta_{1}}$ for the two-port DUT.
- One of the RF sources’ output was phase-shifted.
- The dual SPR measurement procedure was repeated for this phase shift, thus obtaining $\Gamma_{DUT,1,2}^{\theta_{i}}$.
- The phase was shifted a minimum of three times.
- The system of equations in (24) was solved (with least squares if more than three phase measurements were taken), and Equation (26) was then used to compute $S_{21}$ of the DUT.

## 7. Simulated DSPR-VNA Results

To help ensure that all coding, SPR solutions, etc. were correct, we first simulated a DSPR-VNA data collection process using Keysight ADS software, version ADS 2023 Update 2.0. The software has the ability to place multi-port microwave models of exact chips and transmission lines, the power detectors, etc.

We thus used the ADS models of our six-port-circuit design (Section 5.1), combined with idealized (perfectly matched) power detectors at the four power-detector ports. We also simulated two independent RF sources, one of which had a phase shifter attached. The simulation schematic is the same as Figure 4. We then placed several synthetic test DUTs inside the ADS simulation and collected the power meter data. In addition to the DSPR-VNA simulation, the two-port DUT *S*-parameters were measured directly with the ADS software. For this simulation, we selected a frequency range of 0.5 to 3 GHz in 1 MHz steps. The results with a DUT of a high-pass filter (Mini-Circuits SLP2400) are shown in Figure 4.

For the DSPR-VNA, we completed 3 simulations with phase shifts of 0, 30, and 60 degrees for the 2nd RF source. For each phase shift, we used the procedure in Section 6.3 to generate $\Gamma_{1}$ and $\Gamma_{2}$, and then used the SPR-VNA procedure to calculate $S_{11},S_{22}$ and $S_{21}$.

**Figure 8:**
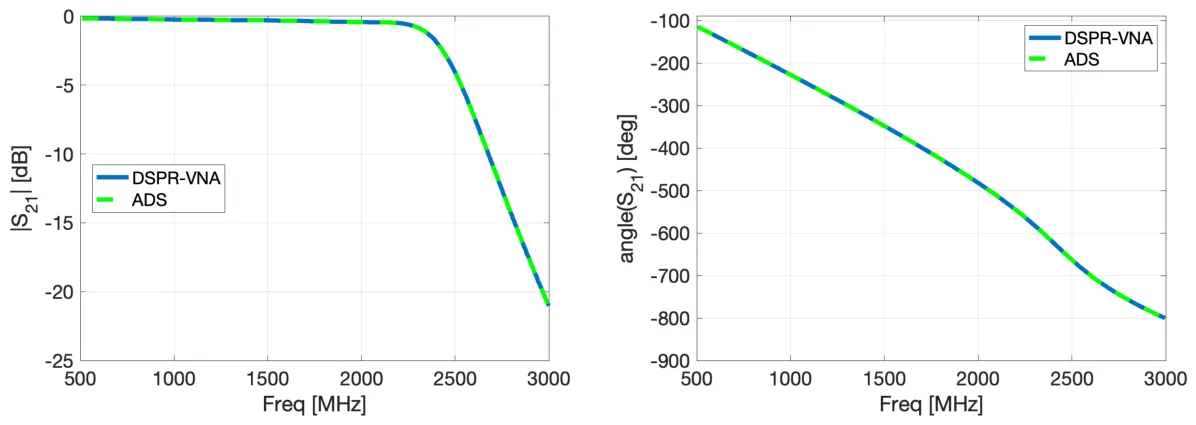
ADS simulation DSPR-VNA $S_{12}$ using SLP2400 DUT. True values (green) vs. the values obtained via simulated DSPR-VNA (blue). Values were the same to within 8 digits.

**Figure 9:**
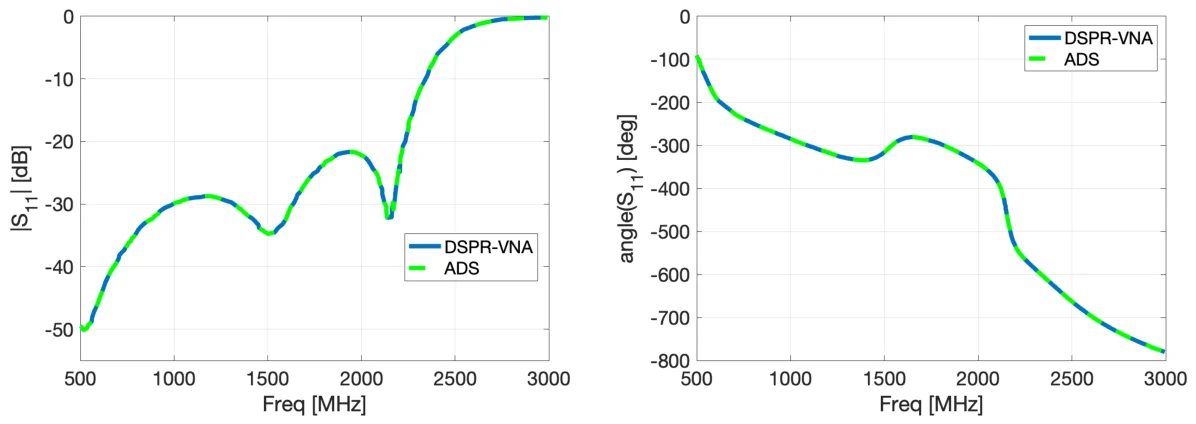
ADS simulation DSPR-VNA $S_{11}$ using SLP2400 DUT. True values (green) vs. the values obtained via simulated DSPR-VNA (blue). The values are the same to within 8 digits.

**Figure 10:**
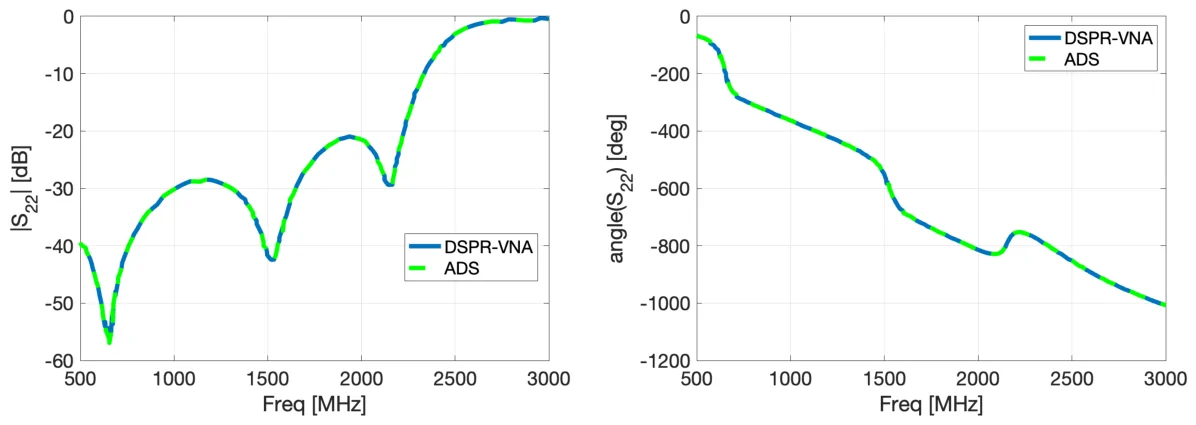
ADS simulation DSPR-VNA $S_{22}$ using SLP2400 DUT.True values (green) vs. the values obtained via simulated DSPR-VNA (blue). The values are the same to within 8 digits.

Figure 8, Figure 9 and Figure 10 show the magnitude and phase of our $S_{12}$, $S_{11}$, and $S_{22}$ results compared with the true $S_{12}$ measured using ADS. The plots show the comparison between ADS- and DSPR-VNA-measured $S_{12}$ magnitude and phase. In all three cases, the true *S*-parameters and DSPR-VNA results are nearly identical, and the results were the same down to 8 digits (nearly −170 dB in log magnitude). The results provide confidence that, in an idealized simulated environment, the theory and implementation of the DSPR-VNA is sound.

## 8. Experimental DSPR-TVNA Results

**Figure 11:**
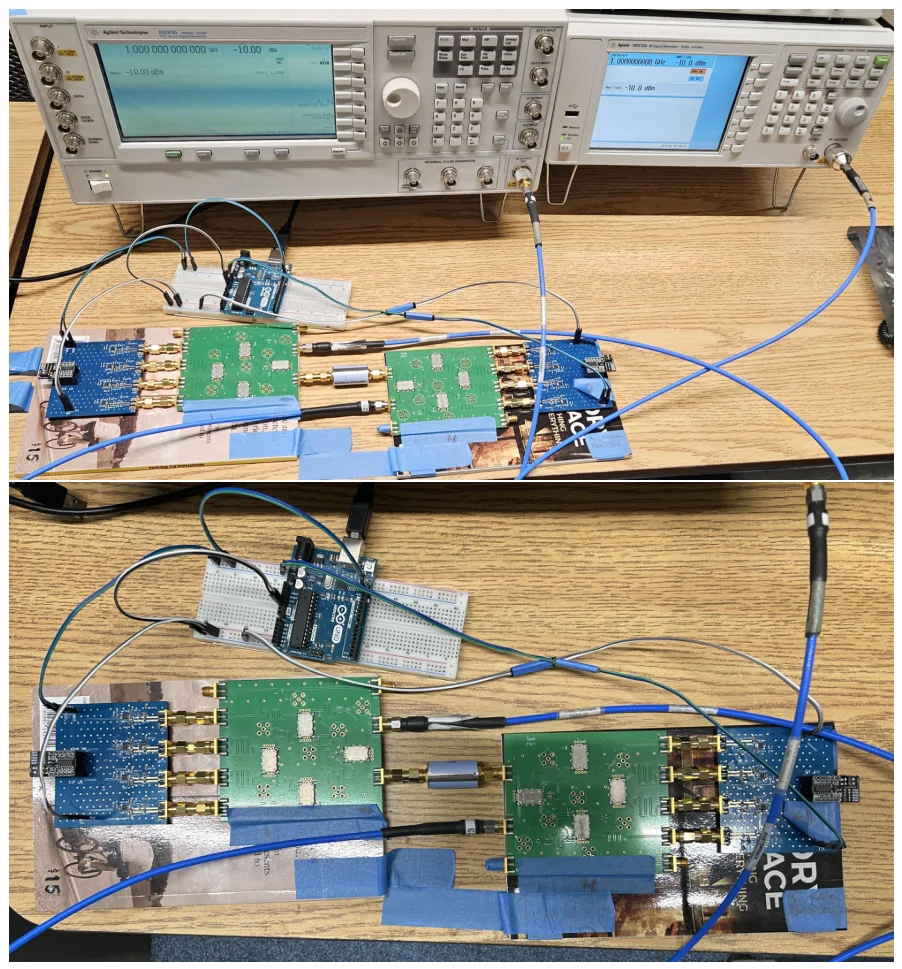
Photo of experimental setup for the DSPR-TVNA. (**Top**): the full setup with RF sources. (**Bottom**): close-up of the two SPRS and the DUT.

Using the hardware described in Section 5.1, we then performed several tests to validate our DSPR-TVNA experimental prototype. Photos of the experimental setup are shown in Figure 11. For a DUT, we present the results from an SLP-1000 low pass filter (Mini-Circuits), measured from 0.6 to 1.2 GHz in 2.5 MHz steps. The ‘true’ *S*-parameters of the DUT were measured with a fully-calibrated Keysight M9802A VNA.

### 8.1. Experimental Results with Phase-Locked RF Sources

The experimental data contain measurement noise, non-linearities in power detectors, limits to the ADC resolution, and other imperfections. Thus, before we use entirely distributed (separated) RF sources, we first present the results with the two RF sources with connected local oscillators. Bench-top RF sources have the ability to obtain an external local oscillator clock signal from another RF source, thus ensuring a near-perfect match between the frequencies outputs. With the two RFs source local oscillators coming from the ‘main’ RF source, we can be sure that they have identical frequency output waves. We thus used the LO output from one RF source to lock in the other source. This process means we are not (at first) using a true distributed SPR-TVNA as the two RF sources are again linked with an RF cable. It did, however, allow us to determine the number of phase shifts required to appropriately solve the system of equations in (24) and to determine an appropriate number of averaging samples to take to reduce noise in our power detector measurements.

Using our experimental apparatus outlined in Section 5.1, we first performed a full set of measurements on the SLP-1000 using a single ADC measurement of each power detector, using 3 different phase shifts: 0, 120, and 240 degrees. The phase shifts were generated via built-in functions of the left-hand RF source shown Figure 11. Three phase shifts is the minimum number to use to solve Equation (24). Our DSPR-VNA processing procedure was then applied to the experimental data.

**Figure 12:**
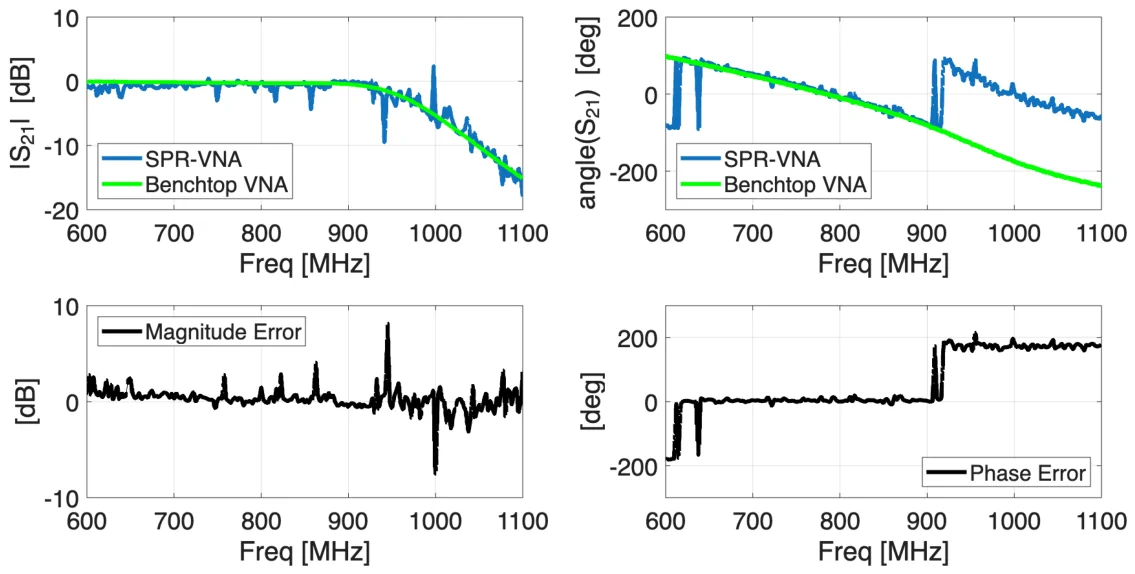
Experimental $S_{21}$ measurement of an SLP1000 low-pass filter with a SPR-TVNA, using 3 phase shifts and one ADC sample per frequency, frequency-locked RF sources, and without phase correction. Commercial VNA measurement (green) vs. SPR-TVNA (blue), and the difference (black).

The results for $S_{21}$ are shown in Figure 12. The top row shows the magnitude and phase of the true $S_{21}$ compared with the DSPR-TVNA, and the bottom row shows the difference. The overall shape of $S_{21}$ is clear, but the results are very noisy. Further, we can see the limitation of the phase measurement with jumps of ±180 degrees seen in multiple places (see Equation (26)).

#### Phase Uncertainty Correction

**Figure 13:**
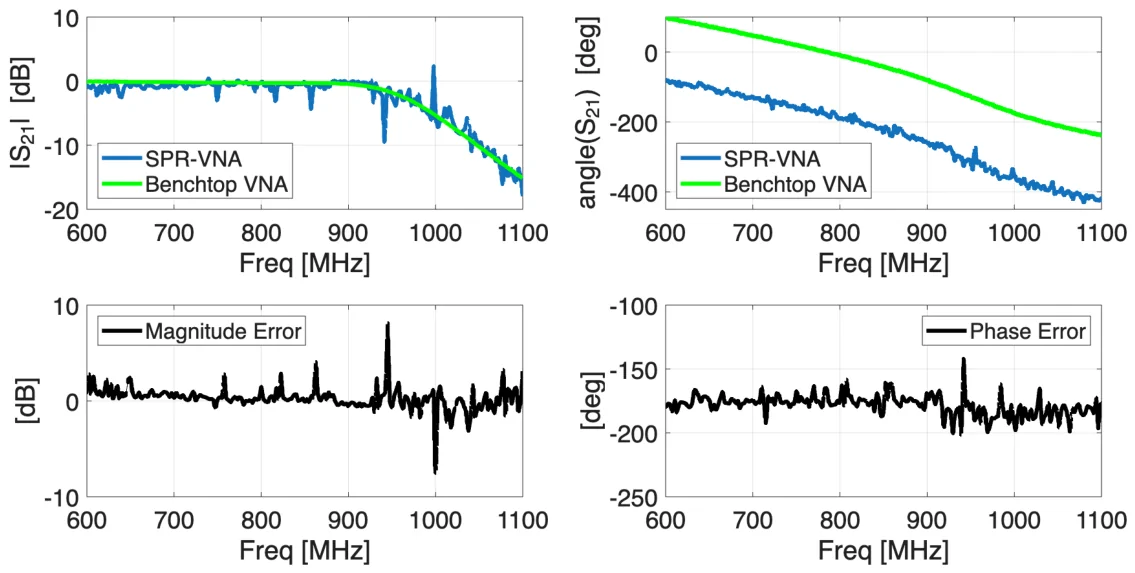
Experimental $S_{21}$ measurement of an SLP1000 low-pass filter with a non-distributed (frequency-locked) SPR-TVNA, with phase unwrapping of large phase jumps. Using 3 phase shifts and one ADC sample per frequency, frequency-locked RF sources. Commercial VNA measurement (green) vs. SPR-TVNA (blue), and the difference (black).

**Figure 14:**
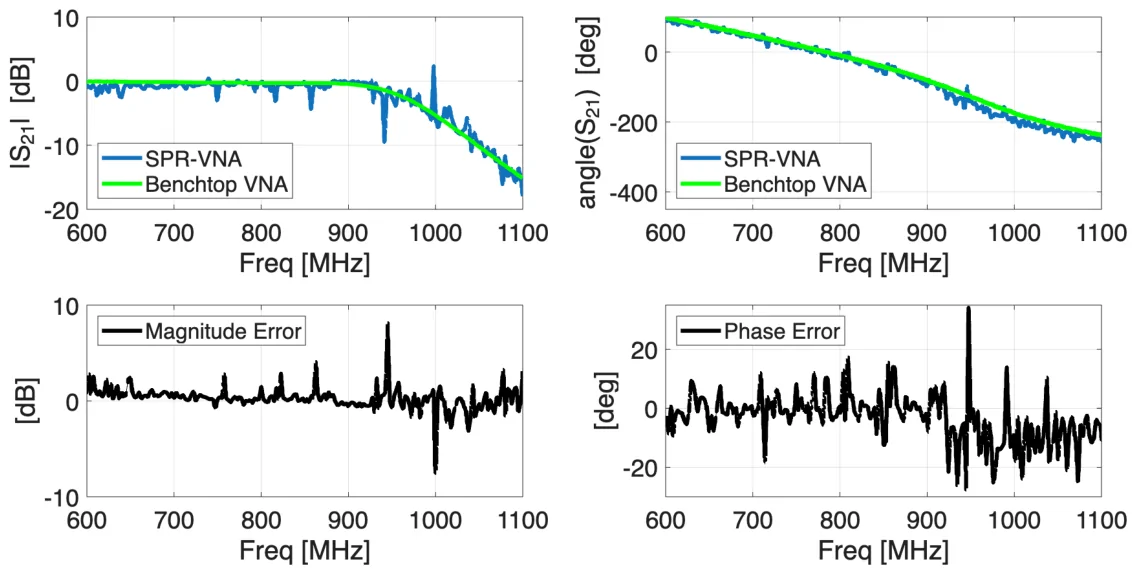
Experimental $S_{21}$ measurement of an SLP1000 low-pass filter with a non-distributed (frequency-locked) SPR-TVNA, with phase unwrapping and correction. Using 3 phase shifts and one ADC sample per frequency, frequency-locked RF sources. Commercial VNA measurement (green) vs. SPR-TVNA (blue), and the difference (black).

To fix the phase jumps, we next performed a phase unwrapping procedure that eliminated jumps of 165 degrees or greater (less than 180 as noise means the jumps are not always equal to 180). These phase-unwrapped results are shown in Figure 13. In these phase-unwrapped results, we can see the fundamental uncertainty of the SPR-VNA process, as the phase is now off by approximately 180 degrees. Using Hoer’s original suggestion that other information be used for the initial ± phase selection [23], we use our knowledge of the true phase via the commercial VNA measurement at the first frequency and perform a 180-degree phase shift. Finally, these phase-corrected results are shown in Figure 14. For all remaining results in this section, we applied the phase-correction procedure.

### 8.2. Noise Reduction Through Multiple Sampling

**Figure 15:**
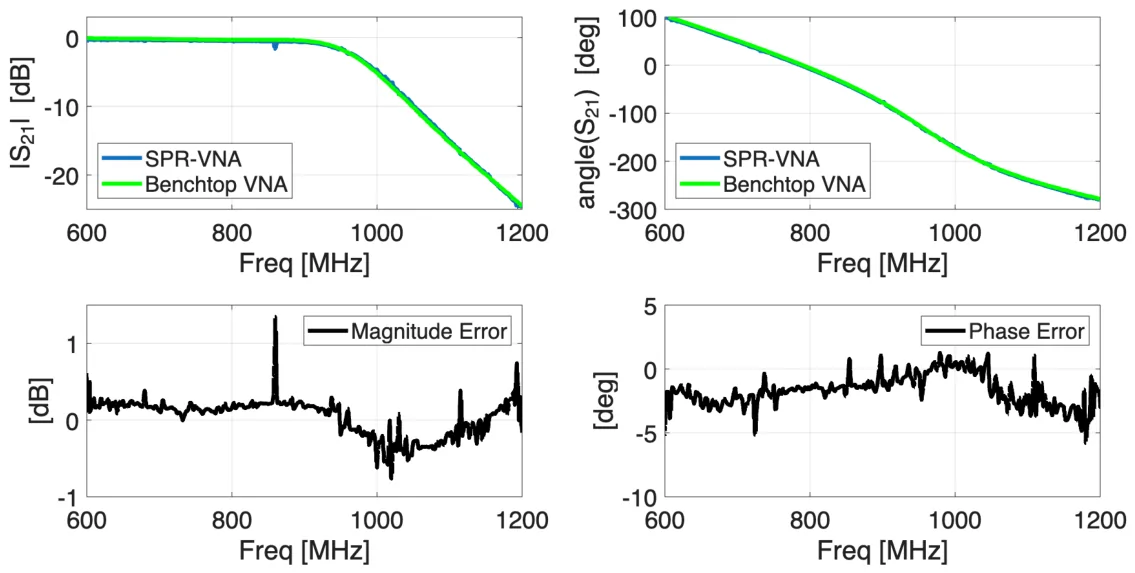
Experimental $S_{21}$ measurement of an SLP1000 low-pass filter with a non-distributed (frequency-locked) SPR-TVNA, with phase unwrapping and correction. Using 3 phase shifts and 10 ADC samples per frequency, frequency-locked RF sources. Commercial VNA measurement (green) vs. SPR-TVNA (blue), and the difference (black).

While the phase of the SPR-VNA result now shows the general shape, it still remains that the results in Figure 14 are very noisy, with maximum errors of 5 dB and 30 degrees. To alleviate this, we performed two extra steps. The first step was to use 10 ADC power measurements for each power detector (eight power detectors, repeated for every frequency). The results with 10-sample averaging are shown in Figure 15. In this case, the noise in the results has been drastically reduced, with maximum errors of 1.5 dB and ≈5 degrees.

**Figure 16:**
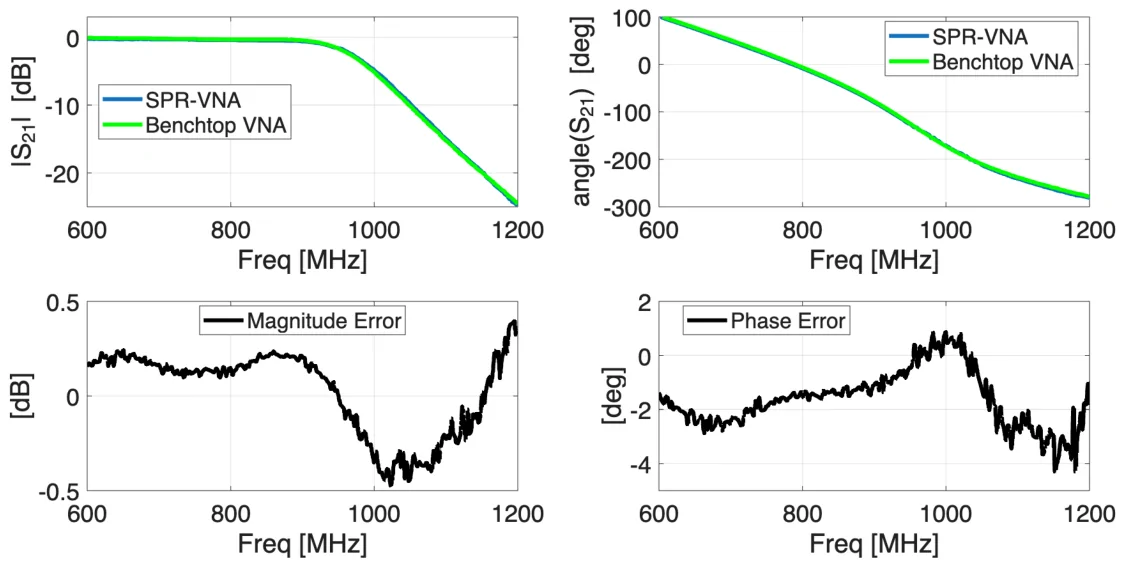
Experimental $S_{21}$ measurement of an SLP1000 low-pass filter with a non-distributed (frequency-locked) SPR-TVNA, with phase unwrapping and correction. Using 10 phase shifts and 10 ADC samples per frequency, frequency-locked RF sources. Commercial VNA measurement (green) vs. DSPR-TVNA (blue), and the difference (black).

Finally, to further improve the results, we added more phase shifts for each measurement, eventually settling on the use of 10 phase shifts per measurement (in steps of 30 degrees). These results for the SLP-1000 are shown in Figure 16. In this case, the maximum magnitude error is lower than 0.5 dB, with maximum phase errors of ≈4 degrees.

**Figure 17:**
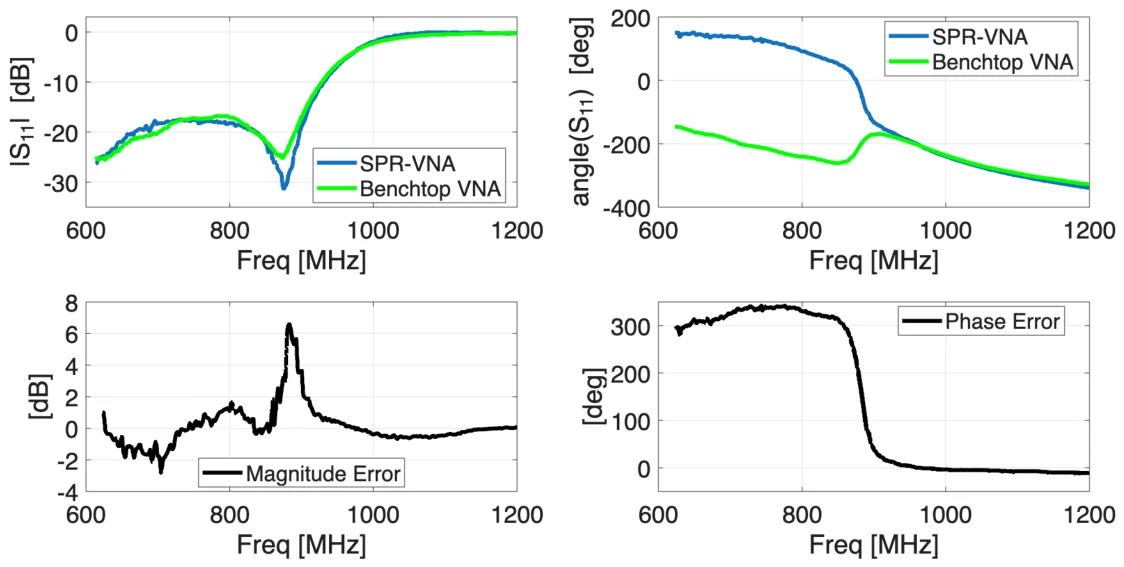
Experimental $S_{11}$ measurement of an SLP1000 low-pass filter with a non-distributed (frequency-locked) SPR-VNA. Commercial VNA measurement (green) vs. SPR-VNA (blue), and the difference (black).

**Figure 18:**
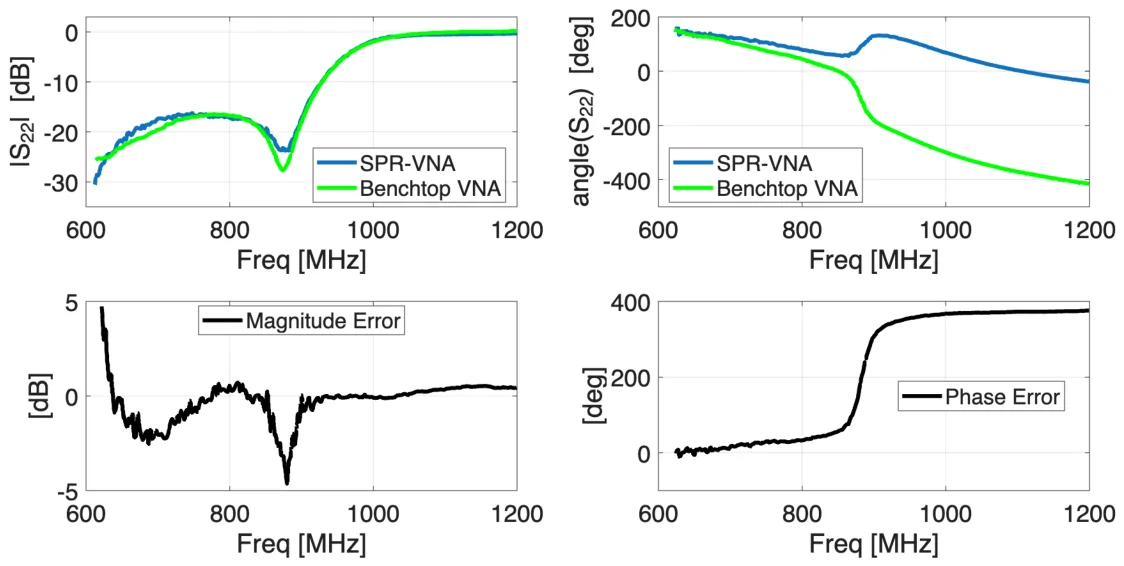
Experimental $S_{22}$ measurement of an SLP1000 low-pass filter with a non-distributed (frequency-locked) SPR-VNA. Commercial VNA measurement (green) vs. SPR-VNA (blue), and the difference (black).

Next, we present the $S_{11}$ and $S_{22}$ data for our (non-distributed) SPR-TVNA results. These results are shown in Figure 17 and Figure 18. Interestingly, the errors are greater in the $S_{11}$ and $S_{22}$ results than in the $S_{21}$ results. It is these errors that caused us to introduce the ‘Transmission-only’ to the name of the experimental device. As $S_{11}$ and $S_{22}$ are computed first (Equation (24)), and $S_{21}$ is subsequently computed from those values, it must be that the errors in $S_{11}$ and $S_{22}$ are canceling out in the calculations in Equation (26). Further comments are presented in Section 9.

### 8.3. Experimental Distributed SPR-TVNA Results

For our final results, we disconnected the two RF sources, thus creating a true distributed SPR-based TVNA. We again used the SLP-1000 as a DUT, and used the 12 phase shift, 10-average data collection procedure from 0.6 to 1.2 GHz in 2.5 MHz steps.

**Figure 19:**
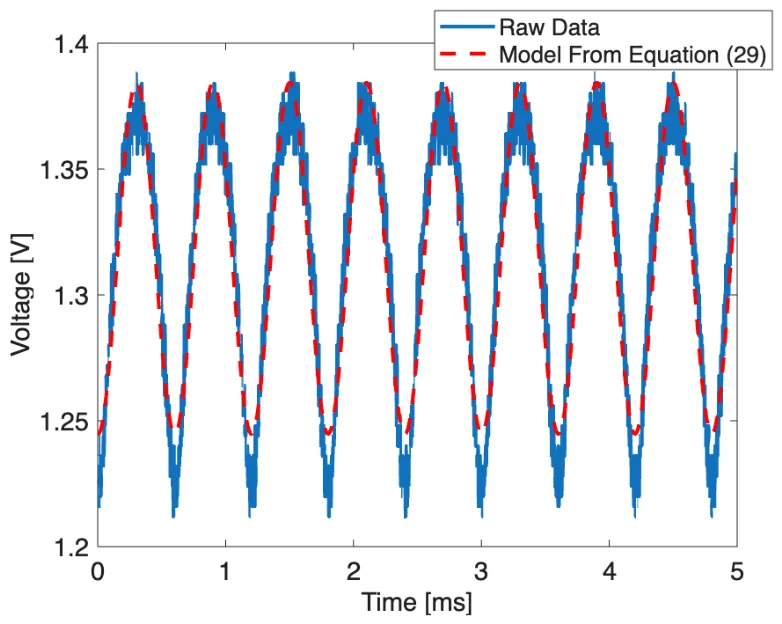
Power detection voltage output levels on Port 4 of SPR-1 with two fully disconnected RF sources and the SLP1000 as DUT. This measured signal (blue) is overlain with a fitted signal based on Equation (29) with $f_{b}$ = 1667 Hz (red), indicating the two RF sources are not outputting at the same frequency.

These initial results are not shown (they simply did not follow the true *S*-parameters in a meaningful way). To diagnose why the complete separation of the two RF sources was causing the measurement process to break down, we probed the output voltage of the AD8313 power detector chips. To do this, we probed the voltage at the power detector output on port 6 of SPR-1 (see port 6 of Figure 2). An example plot of this voltage is shown in Figure 19 (RF sources at 1 GHz, port 6 on the SPR attached to port 2 of the DUT). This signal should, given the theory of the SPR, be a DC voltage. However, there is a clear oscillation of this voltage and the signal is well modeled with a DC-biased |cos(2πft| signal at f=1667 Hz. This indicates that the two RF sources are not outputting at the same frequency, and have a Δf=1667 Hz.

The explanation of this signal is that the two RF sources (with completely independent oscillators) are not outputting the same frequency. This will be further discussed in the next section. As a method of still obtaining reasonable results, we had to consider the limitations of the ADC in our apparatus, which, at 18 bits of resolution, has a maximum sample rate of 3.75 samples/second. Thus, we are unable to capture the signal in Figure 19 and perform more advanced signal analysis. Instead, we attempted a large increase in the number of samples taken by the ADC for each measurement, increasing from 10 ADC samples to 1000 samples. We then took the average of these 1000 samples as the ‘true’ power measurement. Since the sample position of the ADC is effectively random with respect to the oscillating signal, this method should provide the average value of the oscillating signal shown in Figure 19 for each measurement port.

**Figure 20:**
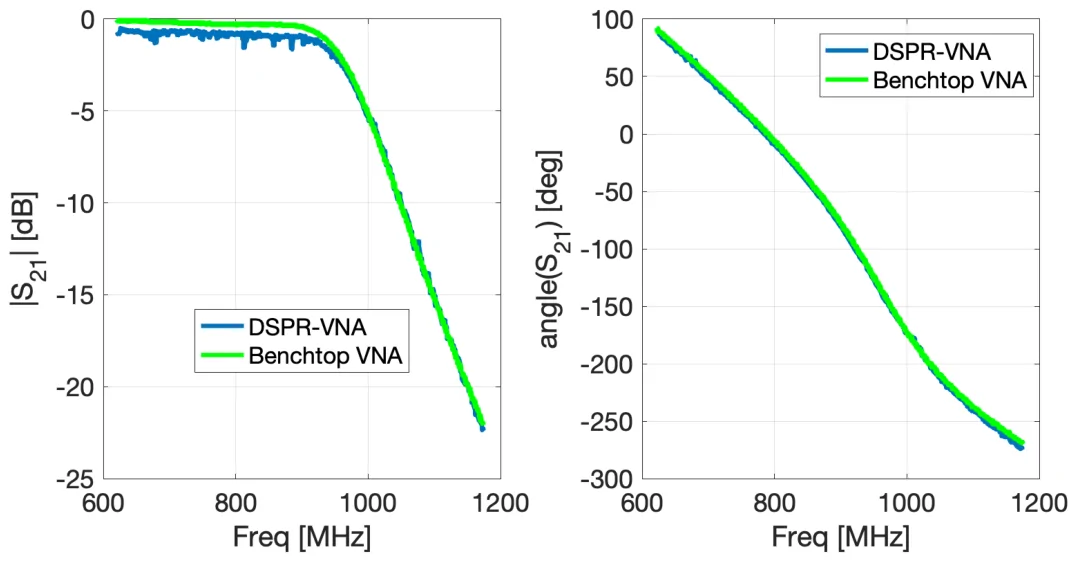
Experimental $S_{21}$ measurement of an SLP1000 low-pass filter with a true *distributed* SPR-TVNA, with phase unwrapping and correction. Using 10 phase shifts and 1000 ADC samples per frequency, completely disconnected RF sources. Commercial VNA measurement (green) vs. DSPR-TVNA (blue).

**Figure 21:**
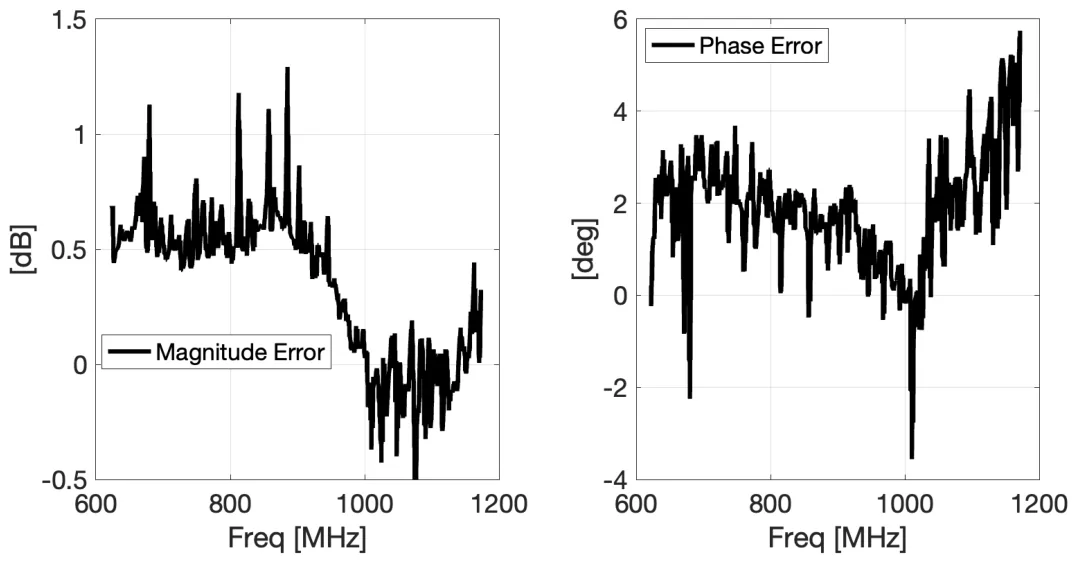
Error in fully distributed DSPR-TVNA $S_{21}$ results of SLP1000.

**Figure 22:**
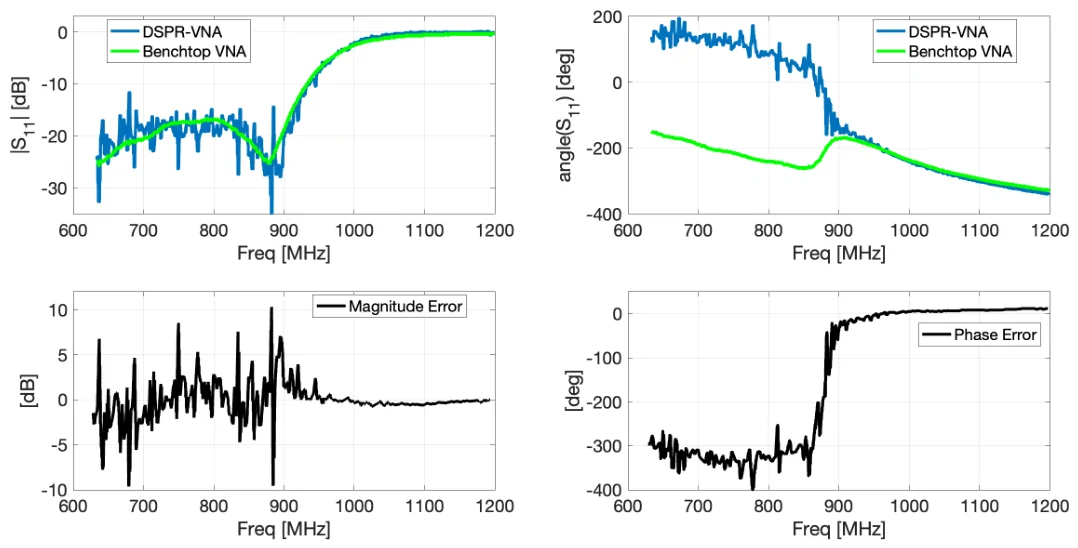
Experimental $S_{11}$ measurement of an SLP1000 low-pass filter with a fully distributed SPR-TVNA. Commercial VNA measurement (green) vs. DSPR-TVNA (blue), and the difference (black).

**Figure 23:**
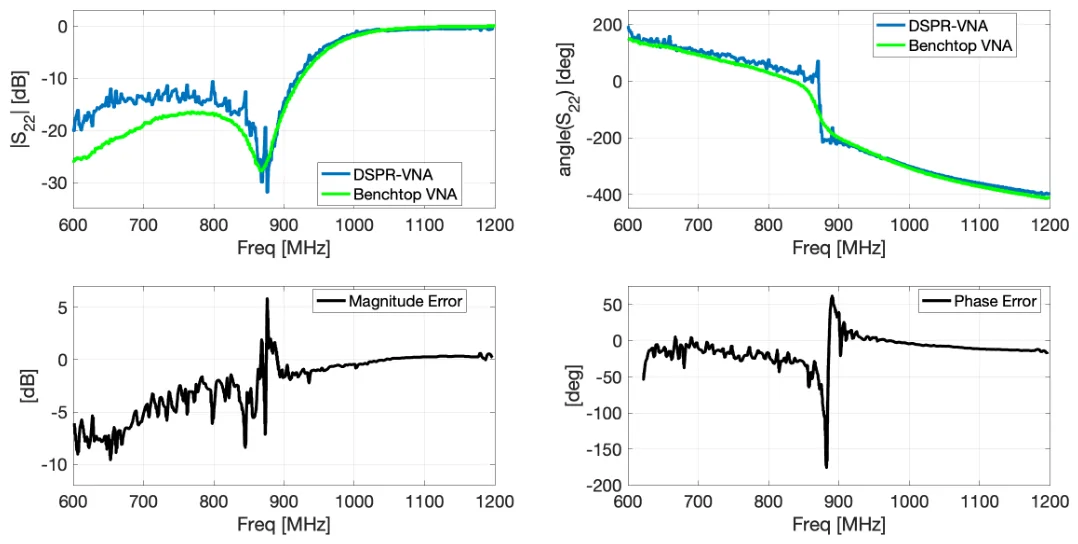
Experimental $S_{22}$ measurement of an SLP1000 low-pass filter with a fully distributed SPR-TVNA. Commercial VNA measurement (green) vs. DSPR-TVNA (blue), and the difference (black).

The final $S_{21}$ results for the experimental DSPR-TVNA, using the 1000 sample-average, are shown in Figure 20. The errors between the true $S_{21}$ and the DSPR-TVNA result are shown in Figure 21, which show maximum errors of 1.5 dB and 6 degrees. Lastly, we show the computed results for $S_{11}$ and $S_{22}$ in Figure 22 and Figure 23. Similar to the non-distributed case, there are significant errors in the $S_{11}$ and $S_{22}$ that are canceled out in the $S_{21}$ calculations.

### 8.4. Analysis of Beat Frequency Signal

The beat frequency signal seen in Figure 19 is the expected signal from the power detector given two slightly different input frequencies of the incoming signals. If we assume that the RF sources are operating at $\omega_{1}$ and $\omega_{2}$, and that at a given power detector, the power of the wave generated by source 1 is $P_{1}$ and the Root-Mean-Square (RMS) power of the wave generated by source 2 is (we note that the exact values of $P_{1}$ and $P_{2}$ will depend on the specifics of the DUT and the SPR) $P_{2}$, then the voltage at the power detector will be a sum of the two waves:

(27) $$v\left(t\right)=v_{1}\left(t\right)+v_{2}\left(t\right)=\sqrt{2P_{1}}cos\left(\omega_{1}t\right)\sqrt{2P_{2}}cos\left(\omega_{2}t+\phi \right),$$

where ϕ is the phase shift between the two sources. The power detector squares this voltage, and then performs low-pass filtering on this signal. After this, the power envelope then becomes:

(28) $$P_{env}\left(t\right)=P_{1}+P_{2}+2\sqrt{P_{1}P_{2}}cos\left(\omega_{b}t+\phi \right).$$

where $\omega_{b}=\omega_{2}-\omega_{1}$ is the beat frequency. The maximum value of this power envelope is ${\left(\sqrt{P_{1}}+\sqrt{P_{2}}\right)}^{2}=P_{1}+P_{2}+2\sqrt{P_{1}P_{2}}$. The minimum value is ${\left(\sqrt{P_{1}}-\sqrt{P_{2}}\right)}^{2}=P_{1}+P_{2}-2\sqrt{P_{1}P_{2}}$.

Key to our approach is that the time *average* power of the power envelope signal $<P_{env}\left(t\right){>}_{t}=P_{1}+P_{2}$, which is our desired power measurement. Thus, averaging the output of the power signal at the detector will result in the desired power measurement.

Finally, we note that since the AD8313 power detector is a log-power detector, and the expected voltage output for these two RF signals is:

(29) $$V_{\mathrm{out}}\left(t\right)=V_{\mathrm{INT}}+U10\log_{10}\left[P_{1}+P_{2}+2\sqrt{P_{1}P_{2}}\cos\left(\omega_{b}t+\phi \right)\right]$$

where $V_{\mathrm{INT}}$ is the intercept voltage, and *U* is the power-detector sensitivity [mV/dB]. An example fit of Equation (29) to the measured power output is shown in Figure 19, where there is a good match between this mathematical model and the measured data.

## 9. Discussion

The differing output frequencies of the two independent RF sources causing a beat frequency at the power-detector output was a major problem for our results. The drift between output frequencies is ultimately caused by differences in the crystal oscillators on board each RF source. While this problem can be solved by averaging the signal over many samples, in the future, this can be solved with either some type of frequency-locking (e.g., a GPS-disciplined crystal oscillator), or by tuning the frequency of each source until the output of the power detectors is a DC signal.

*Acquisition time:* We note that the combination of our slow ADC and the need for 1000-sample averaging for 10 phase shifts leads to lengthy data collection times (many hours for our frequency sweep; the exact time was not tracked). The long acquisition time is primarily driven by the very low sample rate of our current ADC. However, this is easily rectified by using an ADC with a faster sample rate (while maintaining at least 18 bits per sample). Our current ADC was selected assuming a pure DC signal and only a single sample required per frequency, and has a sample rate of only 3.75 samples per second at 18 bits. For example, a 64 ksps ADC would reduce the multi-frequency data acquisition time to less than a minute, and many ADCs sample at much higher rates than even 64 ksps. For measuring grain, sample times of less than one minute are more than sufficient as grain is stable for days to weeks. Further reduction in acquisition time is possible by reducing the 1000-sample average, which can occur with better frequency matching between the two RF sources. We also note that the prototype nature of this device means we did not focus on reducing this acquisition time for this manuscript, and instead focused on reducing the error, as the contribution (and eventual planned application) is not for a fast device.

*Number of averaging samples:* The current selection of 1000 samples to average is somewhat arbitrary—we simply chose a number large enough to reduce the errors to acceptable levels for the current prototype. We did not investigate reducing this number for the prototype as the solutions for reducing this number are the same as above: better-matched frequencies of the RF sources will lead to a (near) DC signal, and a higher sample rate ADC will mean that any beat frequency can be analyzed via other means (e.g., a simple optimization could be used to extract the parameters of the signal in Figure 19 and the average taken, or an analog low-pass filter could be used to eliminate the beat).

It is interesting in our fully-distributed results that the errors for $S_{11}$ and $S_{22}$ are much greater than the errors seen for $S_{21}$. There are also clear 360-degree phase-shift errors (see Figure 17, Figure 18 and Figure 22). This means that the errors in the $S_{11}$ and $S_{22}$ must be offset in Equation (26), along with any errors in the Δ term (which was not shown). In both Equation (26), and in the calculation of Δ (Equation (23)), every time $S_{11}$ and $S_{22}$ appear, they are multiplied together: $S_{11}S_{22}$. Thus, it is the multiplication of their magnitudes and the addition of their phases that must be correct to obtain a correct value for $S_{21}$ (Equation (26)). If the errors offset (e.g., considering magnitudes only), we have $|S_{11}^{meas}\left|=0.9\right|S_{11}^{true}|$ and $|S_{22}^{meas}\left|=\left(1/0.9\right)\right|S_{22}^{true}|$, and then $|S_{11}^{meas}\left|\right|S_{22}^{meas}\left|=\right|S_{11}^{true}\left|\right|S_{22}^{true}|$ and the errors offset. A similar argument may be applied for phases (but with addition).

One of the reasons for errors in our method is that the unknowns of equations in Equation (24) are not actually independent variables. Since Δ is a function of $S_{11}$ and $S_{22}$, this means that even with a low-condition number on the matrix, measurement noise/errors can lead to errors in $S_{11}$ and $S_{22}$. One method of eliminating such errors could be to place matched loads on the opposite port, and directly compute the reflection parameters with each SPR using Equation (13). The $S_{11}$ results (with a hand-placed matched load on port 2) for the SLP1000 are presented in [28], and show errors similar to the errors in our $S_{21}$ results here. Thus, we argue that our distributed SPR-VNA concept can still be called a ‘distributed VNA’, not just a Transmission-Only VNA. However, our current hardware (without the switch and 50 Ω load) is only accurate for transmission-only measurements.

It can perhaps be considered a strength of our method that errors this large can be tolerated while providing more accurate $S_{21}$ results. Future work may look at the ability to use other means of obtaining $S_{11}/S_{22}$ within Equation (26) to improve the accuracy of the $S_{21}$ results.

We also want to note the well-understood sensitivity of six-port reflectometer measurements. The non-linear nature of the SPR problem means that slight changes in the power measurements can cause drastic changes in the obtained S-parameters. For example, we originally used 12 bits of discretization in our ADC, and the results obtained were meaningless (compared to the true S-parameter values). Future hardware iterations will include closer attention to both ADC specifications and noise reduction in the hardware setup.

The results presented here are a deep-dive into a single DUT. Readers who wish to see results for other DUTs are encouraged to read Section 6.4 of ref. [28], where $S_{21}$ measurements of two separated antennas are presented with the phase-locked RF source configuration.

Regarding the selection of the ± in the phase of $S_{21}$, one approach to solve this in grain bin measurements would be to use the phaseless parameterized inversion outlined in [4]. Once an initial model of the bin contents is obtained, a forward solver can be used to obtain the expected phase shift between antennas, which can then be used to select the correct sign.

We finally note that two-port calibration procedure for our distributed SPR-VNA would have likely improved the results. However, we did not perform such calibration due to the lengthy time required to collect data with our prototype presented here.

## 10. Conclusions

This paper presents a novel prototype, proof-of-concept distributed SPR-TVNA with completely independent RF sources working to measure the $S_{21}$s of microwave devices with no direct RF connection between each port of the device. The current hardware can only be called a ‘transmission-only’ VNA due to errors in measuring $S_{11}$ and $S_{22}$. However, the main concern for imaging applications is the through-measurements, and simple hardware changes (i.e., a switch and 50 Ω load) can lead to more accurate reflection measurements [28] Thus, the concept is still called an SPR-VNA. This proves that $S_{21}$ measurements can be taken for ports that are distantly separated in space, leading to new application areas like communication antenna measurement, or removing RF cables from imaging systems like grain bin imaging. Future work will focus on performing faster, more accurate measurements of the SPR-power signals, as well as eliminating $S_{21}$ errors due to the frequency drift between the disconnected RF sources.

## Data Availability

The original contributions presented in this study are included in the article. Further inquiries can be directed to the corresponding authors.

## Appendix A. Short-Open-Load Calibration

In this method, a reciprocal two-port ‘error box’ network is assumed to exist between the SPR and DUT. If we assume that the SPR measures *w*, but the true reflection coefficient is given by $\Gamma_{DUT}$, then the error box introduces the following relationship:

(A1) $$w=\frac{d\Gamma_{DUT}+e}{c\Gamma_{DUT}+1}$$

where *c*, *d* and *e* are the correction coefficients characterizing the error box, and can be obtained by measuring three known loads (an open, a short, and a 50 ohm load), via:

(A2) $$\left[\begin{array}{ccc} -w^{\mathrm{short}\;} & \Gamma_{\mathrm{short}\;} & -1\\ -w^{\mathrm{open}\;} & \Gamma_{\mathrm{open}\;} & -1\\ -w^{\mathrm{load}\;} & \Gamma_{\mathrm{load}\;} & -1 \end{array}\right]\left[\begin{array}{c} c\\ d\\ e \end{array}\right]=\left[\begin{array}{c} -w^{\mathrm{short}\;}\\ -w^{\mathrm{open}\;}\\ -w^{\mathrm{load}\;} \end{array}\right].$$

Once c,d and *e* are obtained, all subsequent SPR measurements are corrected via the following equation:

(A3) $$\Gamma_{DUT}=\frac{e-w}{cw-d}.$$
